# Cancer type-dependent genetic interactions between cancer driver alterations indicate plasticity of epistasis across cell types

**DOI:** 10.15252/msb.20156102

**Published:** 2015-07-30

**Authors:** Solip Park, Ben Lehner

**Affiliations:** 1 EMBL-CRG Systems Biology Research Unit, Centre for Genomic Regulation (CRG)Barcelona, Spain; 2 Universitat Pompeu FabraBarcelona, Spain; 3 Institució Catalana de Recerca i Estudis Avançats (ICREA)Barcelona, Spain

**Keywords:** cancer, epistasis, evolution, genetic interaction networks, tissue specificity

## Abstract

Cancers, like many diseases, are normally caused by combinations of genetic alterations rather than by changes affecting single genes. It is well established that the genetic alterations that drive cancer often interact epistatically, having greater or weaker consequences in combination than expected from their individual effects. In a stringent statistical analysis of data from > 3,000 tumors, we find that the co-occurrence and mutual exclusivity relationships between cancer driver alterations change quite extensively in different types of cancer. This cannot be accounted for by variation in tumor heterogeneity or unrecognized cancer subtypes. Rather, it suggests that how genomic alterations interact cooperatively or partially redundantly to driver cancer changes in different types of cancers. This re-wiring of epistasis across cell types is likely to be a basic feature of genetic architecture, with important implications for understanding the evolution of multicellularity and human genetic diseases. In addition, if this plasticity of epistasis across cell types is also true for synthetic lethal interactions, a synthetic lethal strategy to kill cancer cells may frequently work in one type of cancer but prove ineffective in another.

## Introduction

Most genomic alterations that contribute to cancer affect proteins that are widely expressed and perform functions in most or all cells of the body. However, individual cancer genes often vary dramatically in their importance in different types of cancer (Vogelstein & Kinzler, 2004). Human cancer genome sequencing projects have confirmed this striking heterogeneity of the causal driver alterations across different types of cancer (Ciriello *et al*, 2013; Ding & Wendl, 2013; Kandoth *et al*, 2013a; Zack *et al*, 2013), and indeed, individual genomic alterations vary in their potency to drive cancer when engineered in different cell types in mice (Castellano & Santos, 2011).

Epidemiological modeling in the 1960s predicted that the development of cancer normally involves multiple rate-limiting events (Nordling, 1953; Armitage & Doll, 1954; Cook *et al*, 1969). Subsequently, the discovery of the phenomenon of oncogene cooperation demonstrated that cancer-causing genomic alterations often cooperate with synergistic effects on cell proliferation or survival (Land *et al*, 1983; Ruley, 1983). Cooperation is an example of epistasis (genetic interaction)—the phenomenon whereby the phenotypic effects of combining two mutations differ from the expectation based on the consequences of each mutation alone (Lehner, 2011). Cooperation between two drivers is reflected in the co-occurrence of the two genomic alterations in the same tumors more often than expected by chance. Other cancer driver alterations interact antagonistically, having partially redundant effects giving rise to mutual exclusivity in their occurrence across individual tumors (Ciriello *et al*, 2012).

Epistasis is also an important concept in cancer drug discovery, with substantial efforts directed toward identifying proteins that cause “synthetic lethality” when inhibited in combination with cancer-associated driver or passenger mutations (Hartwell *et al*, 1997; Luo *et al*, 2009; Nijman & Friend, 2013). For example, the synthetic lethal interaction between *BRCA2* mutations and PARP inhibition in breast cancer cells has led to the clinical use of PARP inhibitors in breast cancer patients (Ashworth *et al*, 2011).

An early computational prediction about epistasis in unicellular organisms was that genetic interactions would change depending upon the environmental conditions (Harrison *et al*, 2007). The environmental context dependence of epistasis has been demonstrated in multiple experimental studies (Harrison *et al*, 2007; St Onge *et al*, 2007; Bandyopadhyay *et al*, 2010; Guenole *et al*, 2013; Zhu *et al*, 2014), and epistatic interactions have also been reported to vary across species (Dixon *et al*, 2008; Roguev *et al*, 2008; Tischler *et al*, 2008). Similarly, even within the same cell, how two genetic perturbations interact can differ depending upon the phenotype that is being assayed (Laufer *et al*, 2013).

Using data from more than three thousand human tumors, we show here in a stringent statistical analysis that mutual exclusivity and co-occurrence interactions between cancer driver alterations are frequent, but also that they change in different types of cancer. This plasticity of epistasis across cell types is likely to be a basic feature of genetic architecture in multicellular organisms with implications for designing cancer therapeutics and for understanding evolution and other human genetic diseases.

## Results

### Systematic identification of mutual exclusivity and co-occurrence relationships in human tumors

**Figure 1:**
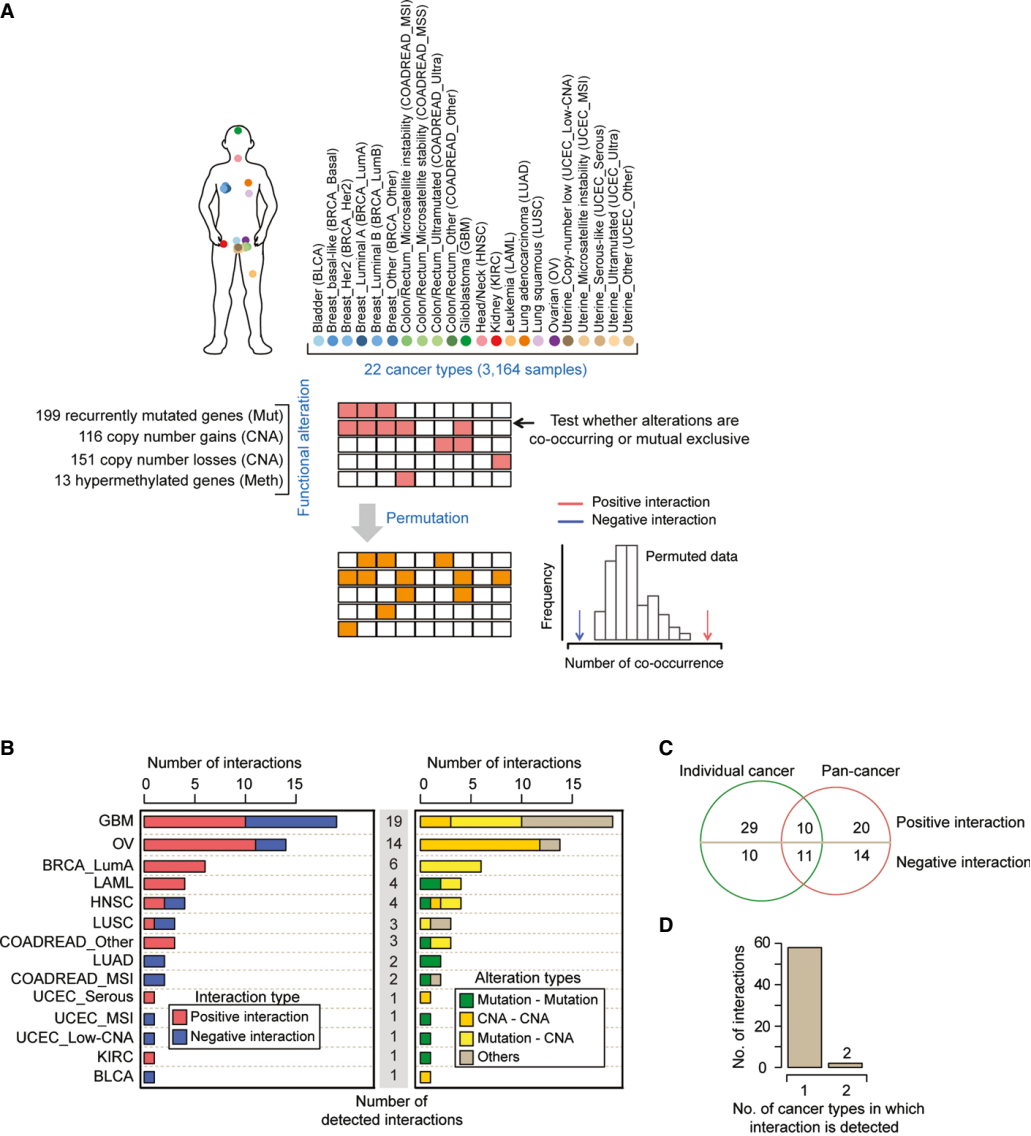
Identification of co-occurrence and mutual exclusivity interactions in different types of cancer Randomization procedure to identify interactions across 22 cancer types.

To identify significant mutual exclusivity and co-occurrence relationships between genomic alterations that contribute to human cancers, we analyzed data from 3,164 tumors of 22 different types studied by The Cancer Genome Atlas (TCGA) consortium (Ciriello *et al*, 2013; Weinstein *et al*, 2013) (Table EV1). We only considered single nucleotide variants (SNVs) in genes significantly mutated in human cancers, recurrent copy number aberrations (CNAs), and a set of recurrent promoter DNA methylation events (see Materials and Methods). To increase statistical power, we restricted our analyses to high-frequency driver events, considering alterations detected in at least 2% of the samples under consideration (Table EV2). We also employed a randomization procedure that accounts for the heterogeneous distribution of alterations across alteration types, samples, and cancer types (Fig1A; see Materials and Methods).

Numbers of interactions, their interaction types (left), and their alteration types (right) identified in analyses of 14 different cancer types in which interactions were detected (FDR = 0.1). The remaining 8 of 22 cancer types had no detected interactions. See FigureEV1 for the same analysis at a stricter FDR (FDR = 0.05).

Overlap between the interactions detected in the pan-cancer analysis and in the analyses of individual cancer types. Of the 39 interactions only detected in the analyses of individual cancer types, 10 were also tested in the pan-cancer analysis. All of the 34 interactions only detected in the pan-cancer analysis were also tested in at least one individual cancer type.

Most interactions were only detected in a single type of cancer.

Considering all 3,164 tumors in a single analysis identified 55 significant relationships between the 86 most recurrent driver alterations (false discovery rate, FDR = 0.1; FigEV1A and B; Table EV3). The interactions were quite evenly balanced between co-occurrence (30 positive interactions) and mutual exclusivity (25 negative interactions). Not surprisingly, more significant interactions were identified for alterations with a higher frequency of occurrence (Spearman’s rank correlation coefficient = 0.34, *P*-value < 0.019; FigEV1C), suggesting that many more interactions will be discovered as more tumors are sequenced and more cancer types are analyzed (FigEV1E–G).

**Figure EV1:**
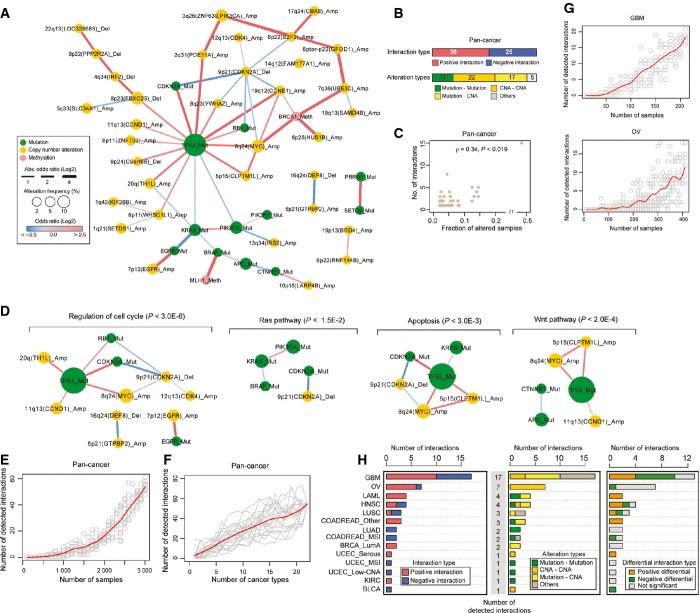
Epistatic interactions detected in a pan-cancer analysis and saturation analysis Pan-cancer epistatic interaction network when the data from the 22 cancer types are analyzed together.

Numbers of genetic interactions, their interaction types, and their alteration types identified in the pan-cancer (FDR = 0.1).

The relationship between alteration frequencies and the number of detected genetic interactions per alteration (node degree) in the pan-cancer network.

Signaling pathway enrichment in the pan-cancer network. The detected genetic interactions are shown for significantly enriched KEGG signaling pathways (hypergeometric *P*-value < 0.05).

Saturation analysis by adding tumors in the pan-cancer analysis. Each point indicates a randomly selected subsample from 3,164 tumors and the red line is a smoothed fit. The number of tumors in the random subset (*x*-axis) and the number of genetic interactions (*y*-axis) are plotted.

Analysis by adding cancer types in the pan-cancer analysis. Each line represents a shuffled ordering of the 22 cancer types. The number of cancer types in the random subset (*x*-axis) and the number of genetic interactions (*y*-axis) are plotted.

Analysis by adding tumors in the two cancer types in which more than 10 genetic interactions were detected. Each point indicates a randomly selected subsample from each cancer type and the red line is a smoothed fit. The number of tumors in the random subset (*x*-axis) and the number of genetic interactions (*y*-axis) are plotted.

Characterization of genetic interactions assigned at a stricter false discovery rate cutoff (FDR = 0.05). Numbers of genetic interactions detected and their interaction types (left), alteration types (middle) in the individual cancer types, and numbers of differential interactions across cancer types (right).

The interactions include intuitive relationships between genes within the same signaling pathways (FigEV1D). For example, interactions were enriched among genes involved in G1/S phase cell cycle regulation (11 interactions among mutation of *TP53*, *CDKN2A*, *RB1*, *EGFR*, and amplifications of 8q24 containing *MYC* and 12q13 where *CDK4* is located) and among genes in the Ras/Raf/MAPK pathway (three interactions among mutation of *BRAF*, *KRAS,* and *PIK3CA*). Previously reported examples of co-occurrence were also detected, including between *PIK3CA* and *KRAS*, and between *CDKN2A* and *TP53* (Kandoth *et al*, 2013a).

### Identification of interactions in individual cancer types

Considering the cancer genome dataset as a single entity biases the discovery of interactions toward those that are conserved across different cancer types or very strong within a subset of cancers. Therefore, we also tested for interactions between recurrent alterations in each of the 22 cancer types considered individually (Fig1A and B). The single cancer type analyses identified 60 interactions between cancer drivers, including 39 not detected in the pan-cancer analysis (Fig1C, FDR = 0.1). For example, an additional six interactions were detected with the *p53* tumor suppressor, and an additional four interactions were detected with deletion of 9p21 where *CDKN2A* is located (Table EV4). Comparing across cancer types, more interactions were detected in cancers in which the median number of samples in which a driver alteration is detected is larger (Spearman’s rank correlation coefficient = 0.64, *P*-value = 0.0013, FigEV2). The interactions include 17 supported by their detection in independent datasets (Table EV4) and at least two that have also been validated using functional assays (Zhao & Vogt, 2008; Etemadmoghadam *et al*, 2013).

**Figure EV2:**
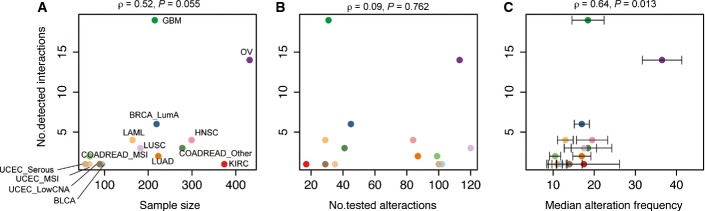
Influence to the number of interactions detected in different cancer types A–C The relationship between number of detected interactions and (A) sample size, (B) number of tested driver alterations, (C) median alteration frequencies of tested driver alterations. Error bars indicate standard errors.

### Interactions between cancer drivers are frequently cancer type specific

**Figure 2:**
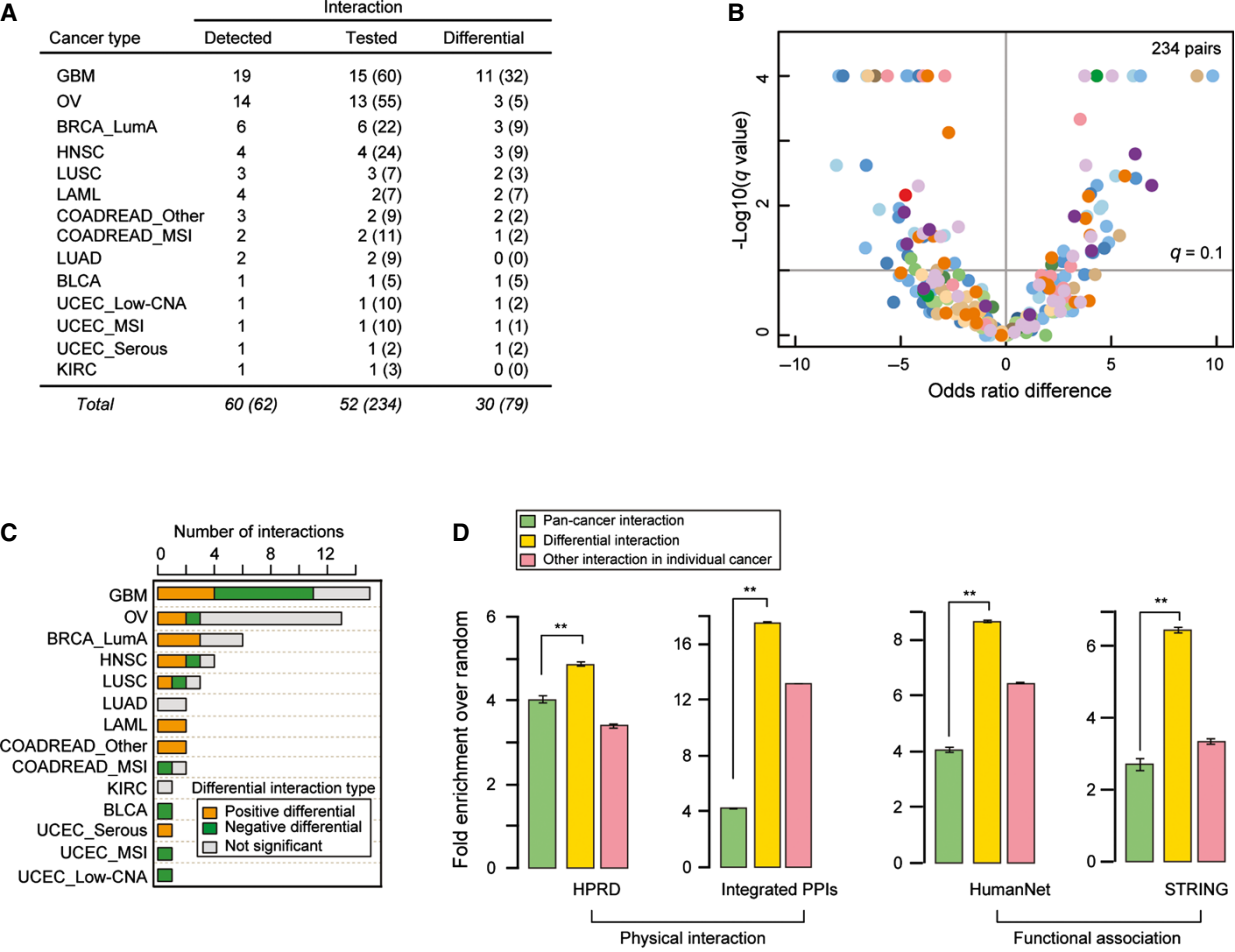
Identifying differential interactions across cancer types and their functional enrichment Fifty-two of sixty driver pairs detected as interacting in one cancer type could be re-tested in at least one other cancer type and 30 of these were detected as interacting differentially (FDR = 0.1). Numbers in parentheses indicate the total number of interactions re-tested and detected, including any redundancy of retesting and detection across different cancer types.

Interestingly, in the single cancer type analyses, more than 90% of the interactions were only detected in a single cancer type (Fig1D; Table EV4). This suggested that how driver alterations interact might be different in different types of cancer. To more directly test this hypothesis, we used an odds ratio (OR) heterogeneity test and permuted data to control for confounders (see Materials and Methods) to evaluate whether each interaction differed between two cancer types. Across all cancer types, we were able to test whether 52 pairs of alterations detected as interacting in one cancer type showed specificity for that type of cancer. Each interaction was re-tested in a median of three other cancer types (mean = 4.3, Fig2A and B).

Volcano plot comparing differences of the log of the odds ratios for the co-occurrence of genomic events in two cancer types (i.e., detected cancer type and compared cancer type). A total of 52 detected interactions were re-tested a total of 234 times in additional cancer types. Color coding is for the cancer type in which the interaction was re-tested, as in Figure1.

Number of significant positive differential, significant negative differential, and non-significantly differential interactions in each cancer type. See Figure EV1 for analyses at a stricter FDR.

Enrichment for physical or functional protein–protein interactions in the pan-cancer analysis (55 pairs) or within each cancer type as differential interactions (30 pairs) or other interactions (30 pairs). Error bars denote 95% confidence intervals (**P-value < 1.0E-3).

**Figure EV3:**
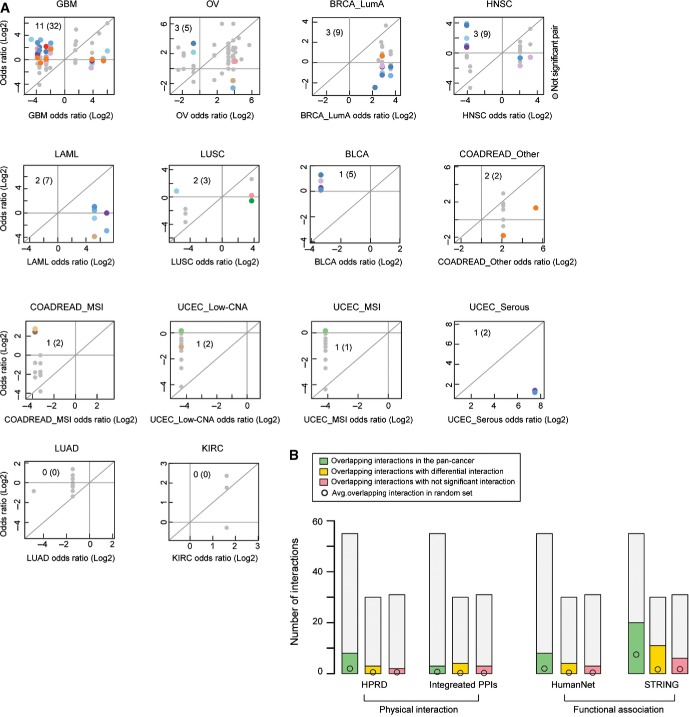
Identifying differential interactions across cancer types and their functional enrichment The odds ratio of co-occurrence in the cancer type in which an interaction is detected (*x*-axis) plotted against the odds ratio of co-occurrence in the compared cancer types (*y*-axis). Colored points are differential interactions (FDR = 0.1) and gray points are non-significantly differential interactions. Color coding is for the cancer type in which the interaction is re-tested, as in Figure1. Inset numbers indicate the non-redundant (redundant) number of differential interactions detected.

This analysis revealed that 57% of the interactions were specific to particular types of cancer (FDR = 0.1, Figs2A and EV3). This included 53% of the examples of co-occurrence and 65% of the examples of mutual exclusivity (Fig2C). Differential interactions were detected both when comparing between cancers from different tissues and when comparing between cancer subtypes from the same tissue (Table EV5).

Numbers of physical and functional protein–protein interactions from four protein–protein interaction sets overlapping the epistatic interactions in each cancer dataset.

**Figure 3:**
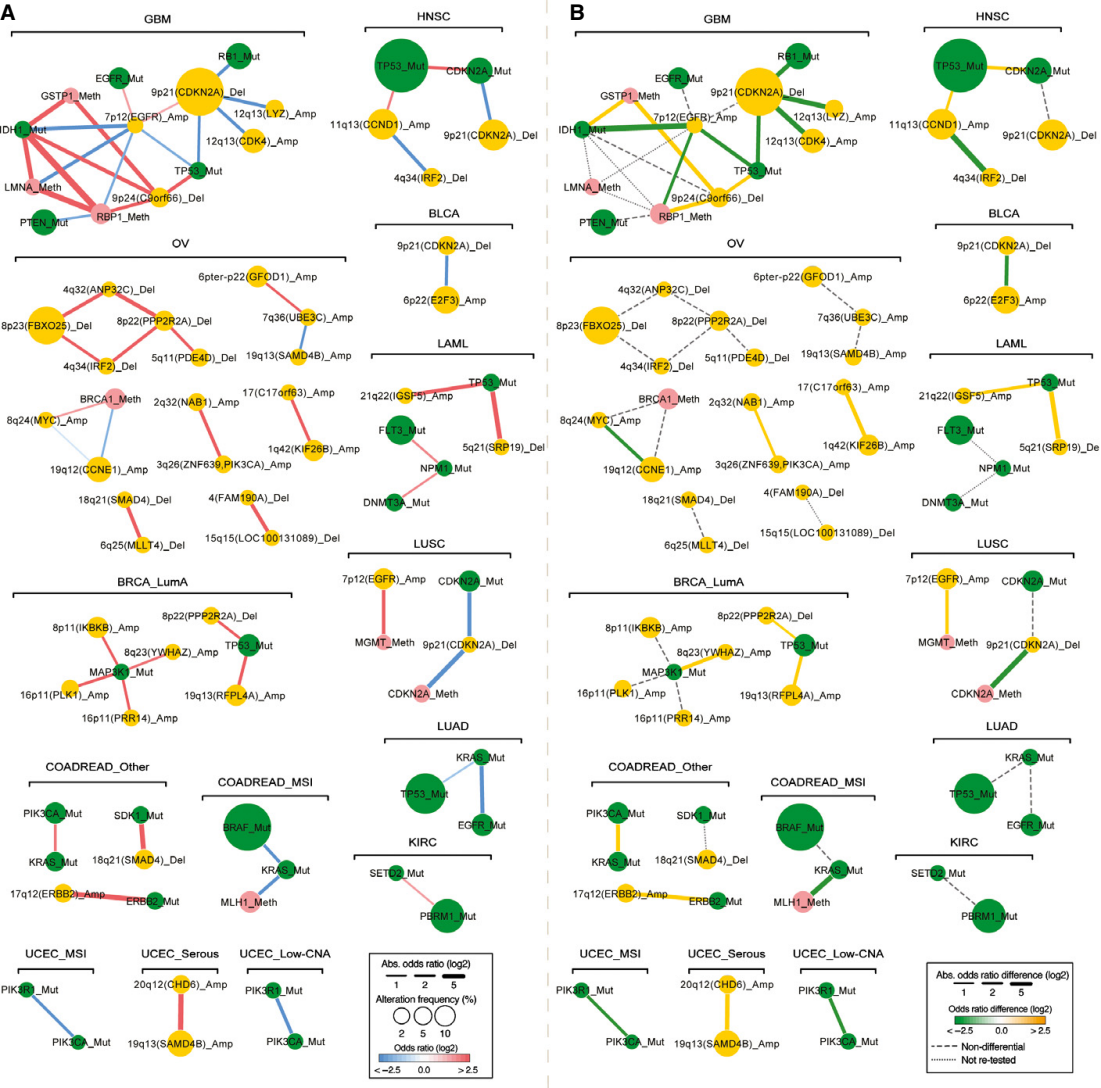
Interaction and differential interaction networks for 14 cancer types In the static network, nodes represent genomic events altered in at least 2% of the samples in each cancer type with their sizes indicating the frequency of alteration and their colors representing the type of alteration. Edge color indicates the interaction type (red: co-occurrence, blue: mutual exclusivity) and width represents the strength of interaction (absolute log of odds ratio).

To illustrate how the interactions change across cancer types, in Figure3 we provide two views of the interaction network detected in each cancer type: a static network indicating the strength of interaction, and a differential network indicating the extent to which each interaction changes in other types of cancer. Together, these networks illustrate how the detected interactions between drivers change across different types of cancer.

In the differential network, edge color corresponds to the type of differential interaction (yellow: higher odds ratio, co-occurrence is significantly stronger in the named cancer type than in another cancer type; green: lower odds ratio, mutual exclusivity is significantly stronger in the named cancer type than in another cancer type; FDR = 0.1). Edge width indicates the strength of differential interaction (absolute difference of the odds ratios between two cancer types). Only the strongest differential odds ratio is shown for each interaction. Cancer types are abbreviated as in Figure1. All networks were drawn using Cytoscape (Smoot *et al*, 2011).

### Cancer type-specific interactions identify functionally related cancer drivers

We compiled datasets of physical and functional interactions between human proteins to investigate how the co-occurrence and mutual exclusivity interactions detected in the tumors relate to previously described relationships between proteins (see Materials and Methods). This revealed that the detected interactions are strongly enriched between genes whose products are known to physically or functionally interact (Fig2D). Interestingly, the interactions showing cancer type specificity are more enriched between genes encoding physically (∼5- to 16-fold) or functionally interacting proteins (∼6- to 8-fold) than the interactions detected when considering all cancer types together (Fig2D, *P* < 3.3E-5; Mann–Whitney test).

### Intra-tumor heterogeneity and unrecognized cancer subtypes do not account for the systematic changes in interactions

**Figure EV4:**
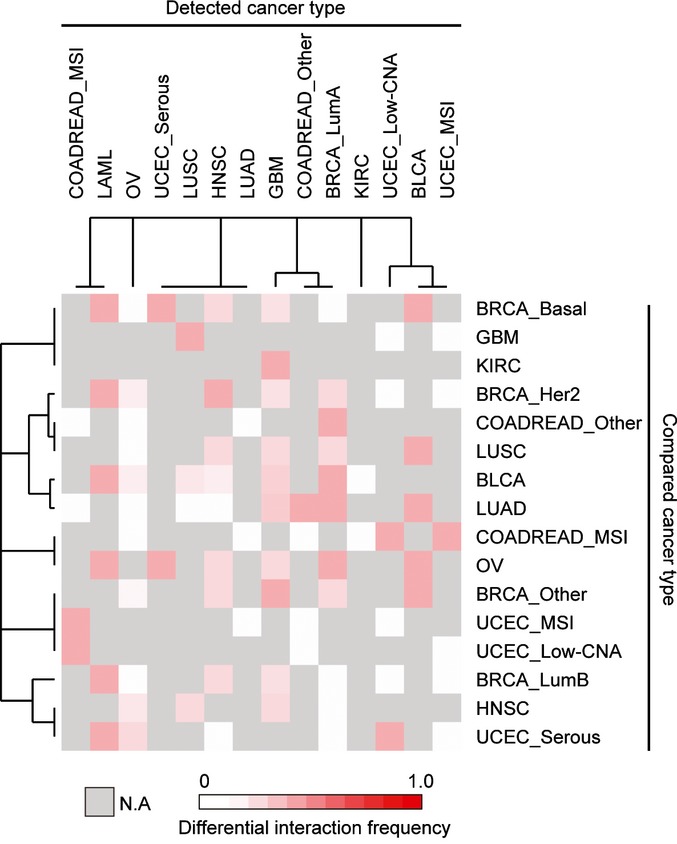
Frequency of detected differential interactions between two cancer types The proportion of tested interactions scored as differential is shown for each pairwise comparison.

Mutual exclusivity and co-occurrence between drivers can be caused by epistasis between the drivers, but they can also potentially be caused by the confounders of intra-tumor heterogeneity or unrecognized cancer subtypes. For example, a high level of intra-tumor heterogeneity in one cancer type could generate false-positive co-occurrence interactions and false-negative mutual exclusive interactions because alterations present in different individual cells within the tumor would wrongly be detected as co-occurring by sequencing. However, if intra-tumor heterogeneity were the cause of the changes in interaction between two cancer types, then the odds ratios of the detected interactions would change in a consistent direction when comparing two cancer types: The ORs should all increase in the more heterogeneous cancer type. This is not the case in our data, with even highly heterogeneous cancers such as glioblastoma having both mutually exclusive and co-occurring relationships that are stronger than in other cancer types (Fig3). Moreover, a similar proportion of differential interactions is detected when comparing between both heterogeneous and non-heterogeneous cancer types (Fig EV4).

In addition to intra-tumor heterogeneity, unrecognized cancer subtypes with subtype-specific driver alterations could also potentially generate false-positive mutual exclusivity and co-occurrence. However, we observe that interactions detected in our analysis are still observed when only considering samples from cancer subtypes that were not explicitly considered in our original analysis (FigEV5). In addition, the strong enrichment for interactions between proteins known to physically or functionally interact (Fig2D) is not consistent with interactions driven by unrecognized subtypes or intra-tumor heterogeneity.

**Figure EV5:**
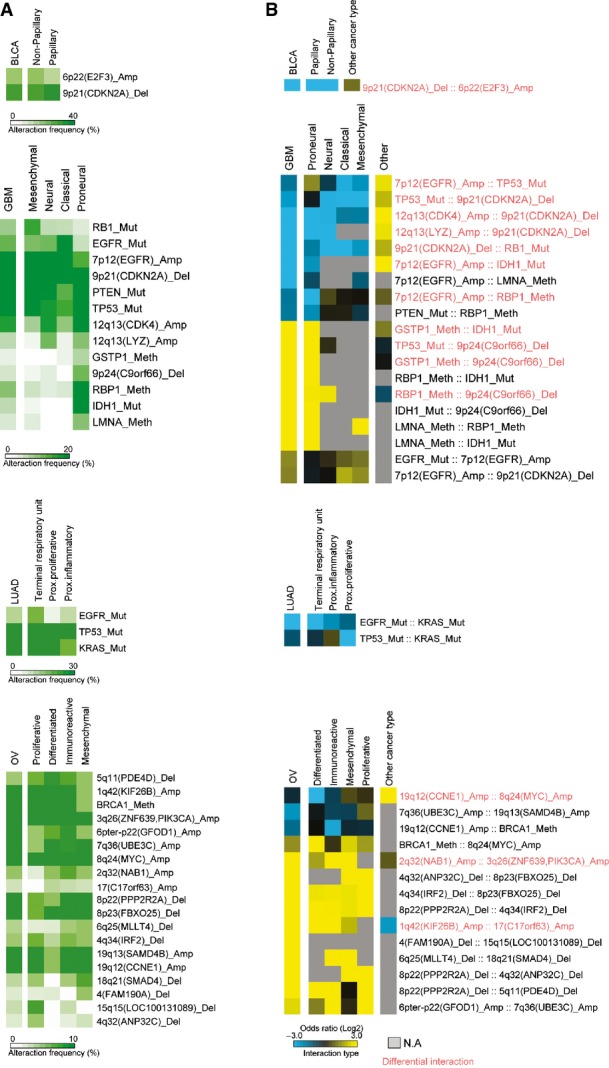
Robustness of interactions across cancer types that were not considered in the main analysis A, B (A) Alteration frequencies of cancer genes across subtypes and (B) odds ratios of the detected interactions in cancer subtypes. Molecular subtypes are as previously defined (Cancer Genome Atlas Research Network, 2011, 2014a,b; Brennan *et al*, 2013).

## Discussion

In this study, we performed a systematic analysis of co-occurrence and mutual exclusivity between cancer driver alterations in different types of cancer. We found that at least half of the interactions between cancer drivers differ in the strength of interaction in different cancer types. This suggests that how genomic alterations interact cooperatively or partially redundantly to driver cancer varies substantially in different cancers. In some cases, these changes in functional relationships across cell types could be due to differences in the precise alterations affecting each driver. In other cases, however, it is likely to be changes in the molecular interaction networks between cell types—for example, changes in feedback or cross talk (Bernards, 2012; Prahallad *et al*, 2012) or the cellular environment—that underlie the changes in potency and epistasis.

Previously, it has been shown in unicellular organisms that epistatic interactions change quite substantially when comparing between two different environmental conditions (Harrison *et al*, 2007; St Onge *et al*, 2007; Bandyopadhyay *et al*, 2010; Guenole *et al*, 2013). This suggests that extensive re-wiring of epistasis across cell types is likely to be a basic feature of the genetic architecture of complex traits.

**Figure 4:**
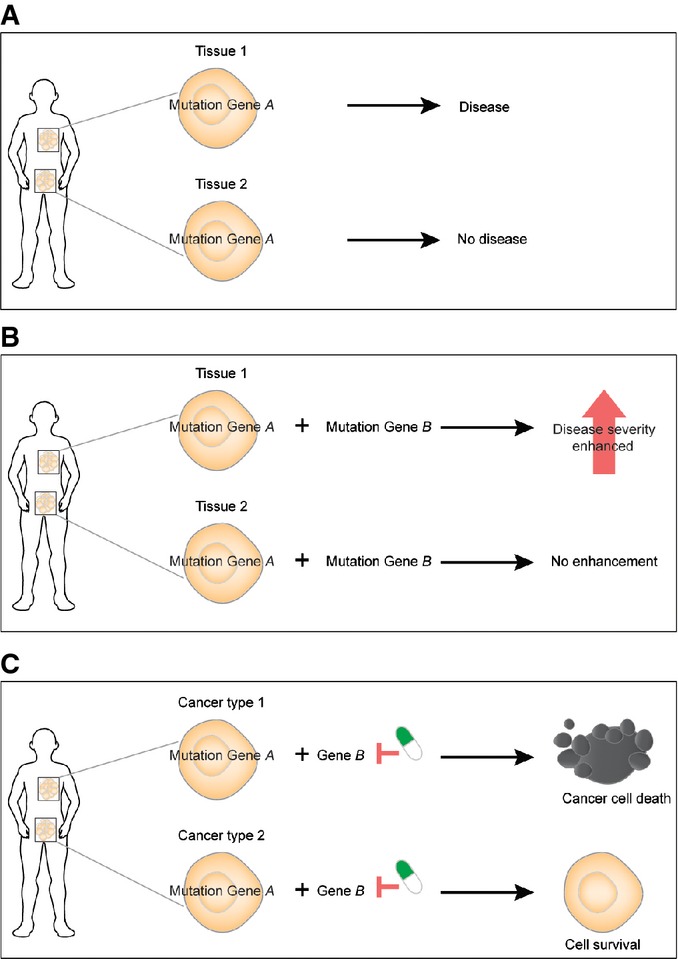
Implications of cell type-specific epistasis for evolution, for other genetic diseases, and for cancer therapy Mutations in universally expressed housekeeping genes can have cell type-specific effects because of cell type-specific epistasis.

The plasticity of epistasis across cell types has important implications for evolution because it allows mutations to alter phenotypic traits in one cell type without necessarily altering traits in other cell types. Moreover, many additional human diseases beyond cancer are caused by mutations in widely expressed housekeeping genes. Although all cells inherit mutated copies of these genes, disease pathology is often limited to a small number of cell types (Lage *et al*, 2008). Our analysis suggests that cell type-specific epistatic interactions will underlie some of the cell type-specific effects of inherited mutations (Fig4A). Our results also predict that the genetic modifier loci of disease-causing mutations will often differ across cell types, which is important to consider for diseases that affect multiple cell types, tissues, or organs (Fig4B).

The modifier loci of a disease gene will change across cell types.

Inhibiting a protein may cause synthetic lethality in combination with a cancer-associated genomic alteration in one type of cancer but fail in another type of cancer.

Epistasis is also an important concept in cancer drug discovery, with large-scale screens being performed to identify genes that are synthetic lethal with cancer-associated genomic alterations (Luo *et al*, 2009; Nijman & Friend, 2013). If the plasticity of epistasis that we detected here is also true for synthetic lethal interactions, then our results have an important take-home message for the exploitation of synthetic lethality in cancer therapy, predicting that particular synthetic lethal strategies will often only prove effective in a limited subset of cancers carrying a targeted vulnerability (Fig4C). Indeed, the available data from large-scale screens and from clinical studies are consistent with this prediction: Synthetic lethal strategies that work *in vitro* in one cell type often fail in another cell type or *in vivo*, and synthetic lethal strategies that are clinically effective in one type of cancer can prove ineffective in a second type (Ashworth *et al*, 2011; Prahallad *et al*, 2012; Lord & Ashworth, 2013). Our results suggest that the set of effective synthetic lethal targets for a defined genomic alteration may vary substantially in different types of cancer, and we propose that drug discovery and evaluation programs should be adjusted accordingly.

## Materials and Methods

### Cancer genome data

We used comprehensive molecular datasets collected across 22 cancer types by the TCGA consortium (Weinstein *et al*, 2013). The number of samples per cancer type varied from 10 (Uterine_Other) to 432 (Ovarian) and the combined dataset comprised 3,164 samples (Table EV1). Hyper-altered samples were excluded (more variant than 3^rd^ quartile + interquartile range × 4.5) (Kandoth *et al*, 2013a). Molecular subtypes were defined in the respective TCGA studies (Cancer Genome Atlas Research Network, 2011, 2012a,b, 2014a,b; Brennan *et al*, 2013; Kandoth *et al*, 2013b).

### Genomic alterations

We considered somatic single nucleotide variants (SNVs), copy number alterations, and DNA methylation events identified by the TCGA consortium (http://cbio.mskcc.org/cancergenomics/pancan_tcga/) (Ciriello *et al*, 2013). In brief, recurrently mutated genes were identified using the MuSiC (Dees *et al*, 2012) and MutSig (Banerji *et al*, 2012) algorithms from whole-exome sequencing data. Somatic mutation calls were assigned to all truncating mutations and to only non-synonymous, single-residue substitutions that existed in hotspots (Ciriello *et al*, 2013). Copy number alterations were determined using GISTIC and the Firehose pipeline (Mermel *et al*, 2011) and filtered for functional alterations using the criteria of concordant mRNA expression as previously reported (Ciriello *et al*, 2013). Levels of DNA methylation were measured as β-values (0, minimal level of DNA methylation; 1, maximal level of DNA methylation), and DNA hypermethylation calls were assigned only to samples with β-values greater than 0.1, filtering for concordant mRNA expression changes. A total of 479 functional alterations, consisting of 199 recurrently mutated genes, 151 copy number losses, 116 copy number gains, and 13 epigenetically silenced genes, were analyzed in this study (Table EV2).

### Candidate genes within copy number alterations

To further prioritize individual genes within chromosomal copy number alterations, we analyzed the concordance between mRNA expression level and copy number changes across samples for cancer types that have differential interaction partners. For each region, we classified samples into altered (amplified or deleted) and not altered and tested for a difference in expression using the Mann–Whitney test. mRNA expression data were obtained from cBioportal (Gao *et al*, 2013) and were log2-transformed. Genes within chromosomal events were sorted according to the FDR *q* values (FigEV6).

**Figure EV6:**
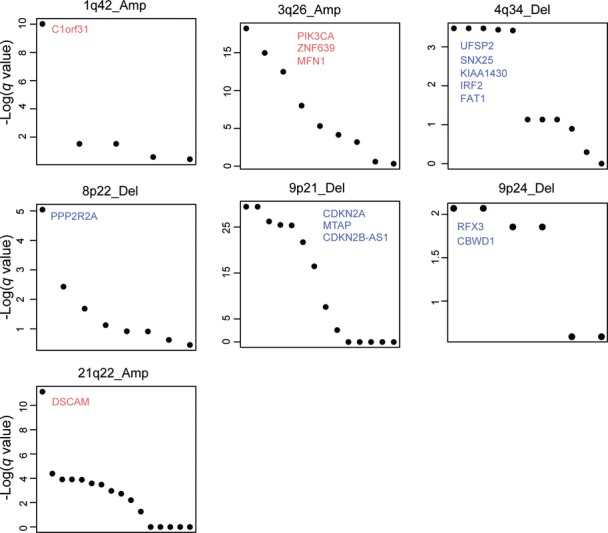
Candidate driver genes within copy number alterations Seven recurrent gains and losses on chromosomes 1q, 3q, 4q, 8p, 9q, and 21q with differential interaction partners contain multiple genes. Genes are ranked within each copy number alteration using the FDR *q* values from a test for a difference in mRNA expression between altered and non-altered samples (Mann–Whitney test).

### Detection of interactions

To determine the significance of the co-occurrence or mutual exclusivity of a pair of functional events, we applied a permutation strategy that controls for the mutational heterogeneity within and across tumor samples. We used the permatswap function in the R package vegan (http://vegan.r-forge.r-project.org/) to produce permutated genomic alteration matrices that maintain the total number of alterations for each alteration across samples as well as the total number of alterations per sample, considering copy number alterations, somatic mutations, and DNA hypermethylations as separate classes. We permuted genomic events for each cancer type separately to control for any biases in alteration frequencies in the different cancer types. A total of 10,000 permutations were performed, and the proportion of permutations in which the observed co-occurrence was higher (*P*_co_) or lower (*P*_me_) than in the real data was taken as an empirical *P*-value. In the pan-cancer analysis, permutations were also performed within each cancer type separately and then the numbers of co-occurrences were summed across cancer types. We only tested for interactions between copy number alterations on different chromosomes to avoid the confounding influence of linkage. To correct for multiple hypothesis testing, the minimal *P*-values of *P*_co_ and *P*_me_ were converted to FDR using the method of Benjamini and Hochberg (1995) with the p.adjust function in R.

### Analysis of differential interactions

To test whether interactions differ between cancer types, we considered 52 pairs of alterations that were detected as significant interactions in one cancer type and that were also individually altered in at least 2% of the samples of at least one other cancer type. In each cancer type, we quantified the co-occurrence of the two genomic events using a 2 × 2 contingency table and the odds ratio as follows:

**Table:** 

**Cancer type *i***	**Altered frequency in Gene 2**	**Non-altered frequency in Gene 2**
Altered frequency in Gene1	CO_*i*_	B only_*i*_
Non-altered frequency in Gene 1	A only_*i*_	Neither_*i*_

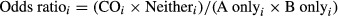

**Table:** 

**Cancer type *j***	**Altered frequency in Gene 2**	**Non-altered frequency in Gene 2**
Altered frequency in Gene 1	CO_*j*_	B only_*j*_
Non-altered frequency in Gene 1	A only_*j*_	Neither_*j*_

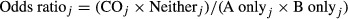

0.5 was added to each frequency when calculating odds ratios to avoid division by zero frequencies. We then performed Tarone’s test for the heterogeneity of odds ratios (ORs) using the R package metafor (http://cran.r-project.org/web/packages/metafor/). To control the mutational heterogeneity between cancer types, we compared the observed Tarone’s heterogeneity test statistic (*T*_obs_) with the statistic from 10,000 permuted datasets (*T*_random_). Permutations were performed as described above, and the proportion of permutations in which *T*_random_ was greater than *T*_obs_ was used as an empirical *P*-value and corrected for multiple hypothesis testing using the method of Benjamini and Hochberg.

### Saturation analysis

To explore whether the number of detected interactions is approaching saturation or not, we performed two different saturation analyses: first, down-sampling within each cancer type (only including those with more than 10 detected interactions); and second, down-sampling of the number of different cancer types for the pan-cancer analysis. In the down-sampling within each cancer type, the sizes of subsampling were defined to sample regularly (by adding 10 samples each time) in the interval from 10 tumor samples to the total number of samples within each cancer type, and this process was repeated 10 times. In the down-sampling of the pan-cancer, we added 100 samples each time in the interval from 100 tumor samples to the final number (3,000) and repeated this process 10 times. To evaluate the effect of adding various different cancer types, we defined 22 random orderings of the 22 cancer types. For each ordering, we sequentially added a different cancer type according to the random order. In each random set, 10,000 permutations were performed.

### Functional evaluation of interactions

We compared the interaction networks to physical and functional protein–protein interactions from four different sources: HPRD (Human Protein Reference Database) v.9 (Keshava Prasad *et al*, 2009), an integrated and filtered set of protein–protein interactions (Park *et al*, 2011), STRING (Search Tool for the Retrieval of Interacting Genes/Proteins) v.9 (Franceschini *et al*, 2013), and HumanNet (Human gene functional interaction network) v.1 (Lee *et al*, 2011). HPRD is a manually curated protein–protein interaction database and the integrated and the filtered protein interaction set merges seven existing interaction databases (the Biomolecular Interaction Network Database, the Molecular Interaction Database, the Database of Interacting Proteins, IntAct, BioGRID, Reactome, and the Protein–Protein Interaction Database) after excluding low-confidence protein–protein interaction pairs, as described (Park *et al*, 2011). STRING integrates various biological datasets such as gene expression and high-throughput experiments. We only considered STRING interactions with confidence scores greater than 700 and excluded experimentally validated protein–protein interactions (evidence score of experiment is higher than 400). HumanNet is a probabilistic functional gene network built by integrating 21 different types of evidence. We took gene pairs with an interaction score greater than 2.0. For copy number alterations, genes in the altered regions were reported in a previous study (Ciriello *et al*, 2013). We assigned an edge between two genomic alterations when at least one protein encoded in the first genomic alteration has a physical or functional protein–protein interaction with a protein encoded in the second alteration. To measure the enrichment for protein–protein interactions shown in Figure2D, we randomly selected the same number of detected interacting pairs from recurrent altered genes (at least 2% of samples from the cancer types) 1,000 times. Enrichment for protein–protein interactions was calculated as follows:

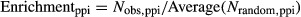
where: *N*_obs,ppi_ = total number of protein–protein interactions among the detected pairs; *N*_random,ppi_ = total number of protein–protein interactions among the randomly selected pairs.

### Change in interaction between two types of cancer

The change in interaction between two types of cancer was quantified as the change in the log of the odds ratio.

## Supporting Information

**Expanded View Figures PDF**

**Table EV1**

**Table EV2**

**Table EV3**

**Table EV4**

**Table EV5**

**Review Process File**
