# Supplementary material for: Cancer type-dependent genetic interactions between cancer driver alterations indicate plasticity of epistasis across cell types
Source: Mol Syst Biol. 2015 Jul 30;11(7):824. doi: 10.15252/msb.20156102 (PMC4547852; doi:10.15252/msb.20156102)
Supplement: Supplementary file 1 [file msb0011-0824-sd1.pdf]

Expanded View Figures

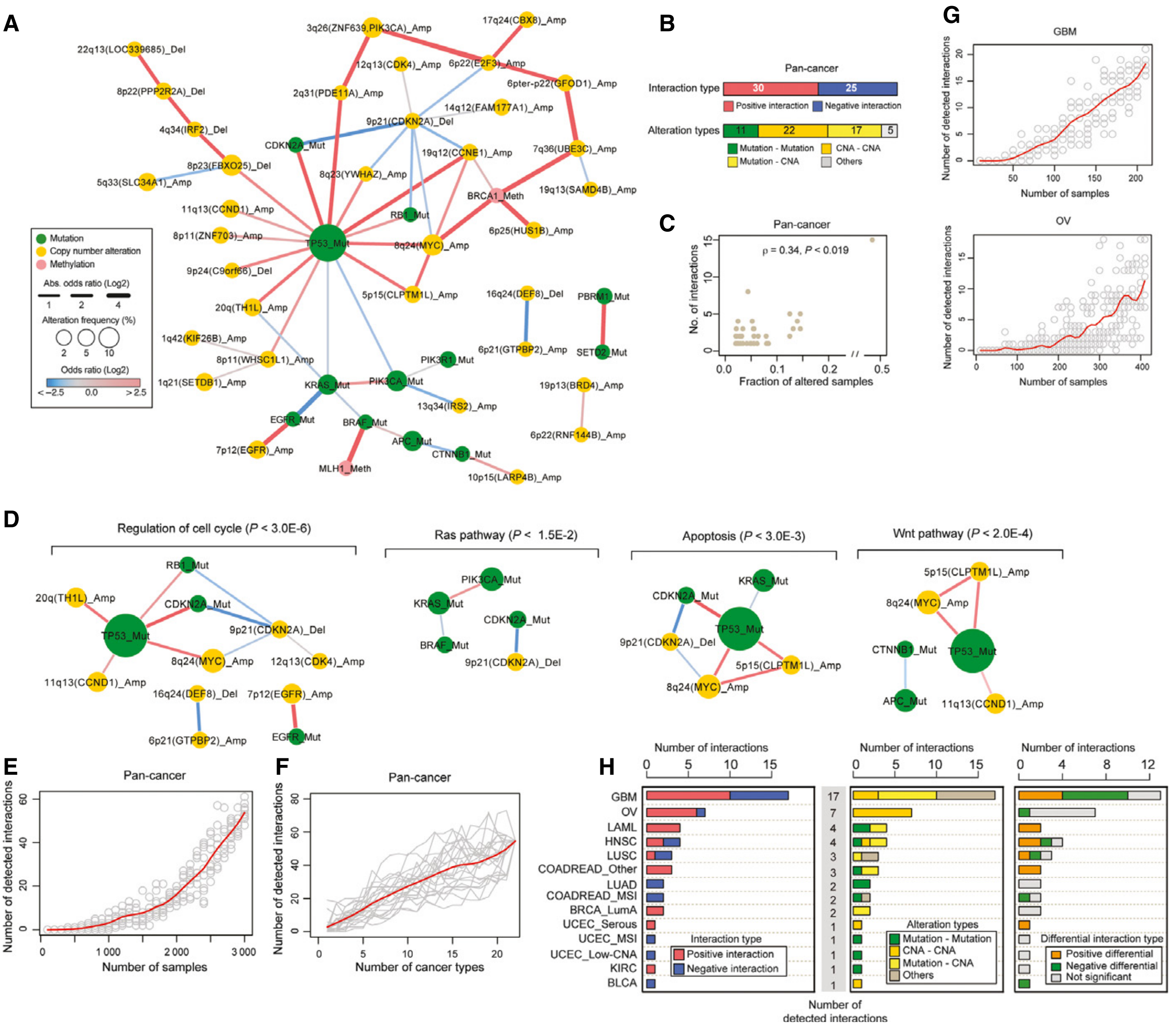

Figure EV1. Epistatic interactions detected in a pan-cancer analysis and saturation analysis.

A Pan-cancer epistatic interaction network when the data from the 22 cancer types are analyzed together.

B Numbers of genetic interactions, their interaction types, and their alteration types identified in the pan-cancer (FDR = 0.1).

C The relationship between alteration frequencies and the number of detected genetic interactions per alteration (node degree) in the pan-cancer network.

D Signaling pathway enrichment in the pan-cancer network. The detected genetic interactions are shown for significantly enriched KEGG signaling pathways (hypergeometric P-value < 0.05).

E Saturation analysis by adding tumors in the pan-cancer analysis. Each point indicates a randomly selected subsample from 3,164 tumors and the red line is a smoothed fit. The number of tumors in the random subset (x-axis) and the number of genetic interactions (y-axis) are plotted.

F Analysis by adding cancer types in the pan-cancer analysis. Each line represents a shuffled ordering of the 22 cancer types. The number of cancer types in the random subset (x-axis) and the number of genetic interactions (y-axis) are plotted.

G Analysis by adding tumors in the two cancer types in which more than 10 genetic interactions were detected. Each point indicates a randomly selected subsample from each cancer type and the red line is a smoothed fit. The number of tumors in the random subset (x-axis) and the number of genetic interactions (y-axis) are plotted.

H Characterization of genetic interactions assigned at a stricter false discovery rate cutoff (FDR = 0.05). Numbers of genetic interactions detected and their interaction types (left), alteration types (middle) in the individual cancer types, and numbers of differential interactions across cancer types (right).

© 2015 The Authors

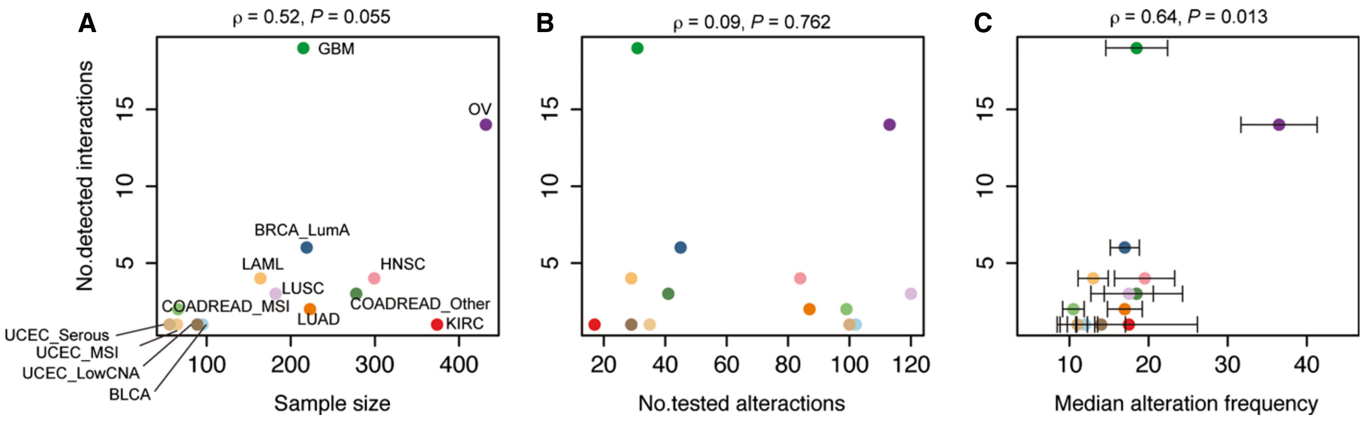

Figure EV2. Influence to the number of interactions detected in different cancer types.

A–C The relationship between number of detected interactions and (A) sample size, (B) number of tested driver alterations, (C) median alteration frequencies of tested driver alterations. Error bars indicate standard errors.

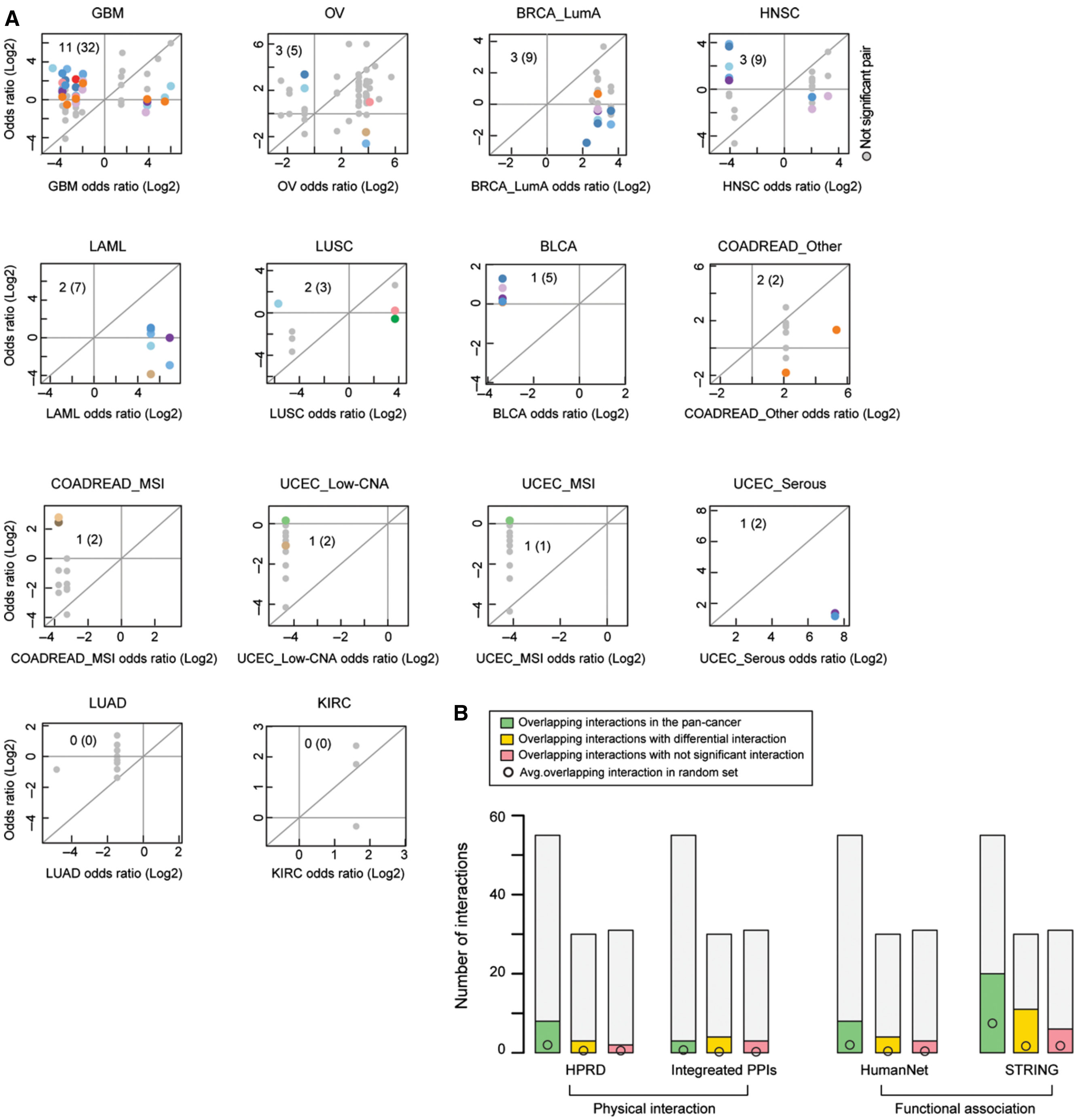

Figure EV3. Identifying differential interactions across cancer types and their functional enrichment.

A The odds ratio of co-occurrence in the cancer type in which an interaction is detected (*x*-axis) plotted against the odds ratio of co-occurrence in the compared cancer types (*y*-axis). Colored points are differential interactions (FDR = 0.1) and gray points are non-significantly differential interactions. Color coding is for the cancer type in which the interaction is re-tested, as in Figure 1. Inset numbers indicate the non-redundant (redundant) number of differential interactions detected.

B Numbers of physical and functional protein–protein interactions from four protein–protein interaction sets overlapping the epistatic interactions in each cancer dataset.

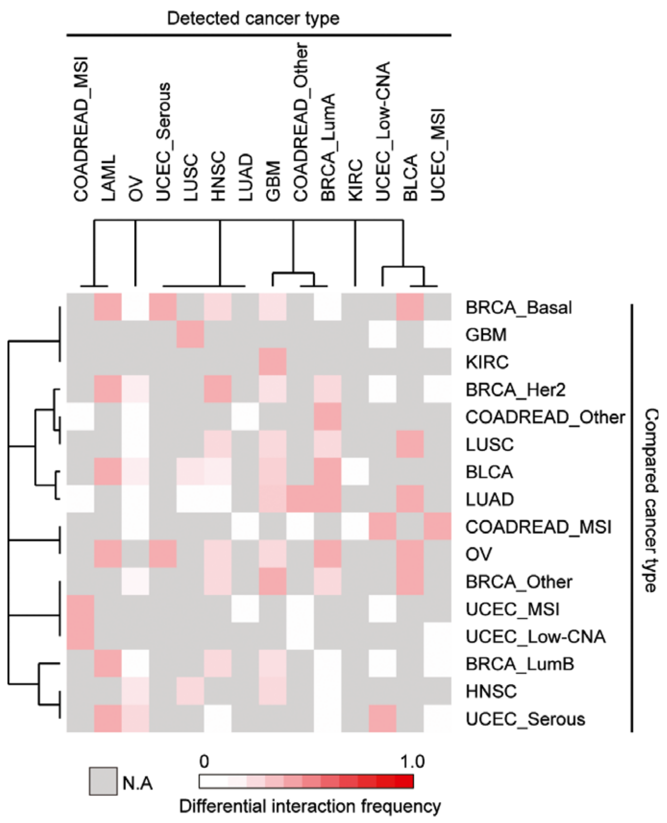

Figure EV4. Frequency of detected differential interactions between two cancer types.
The proportion of tested interactions scored as differential is shown for each pairwise comparison.

© 2015 The Authors

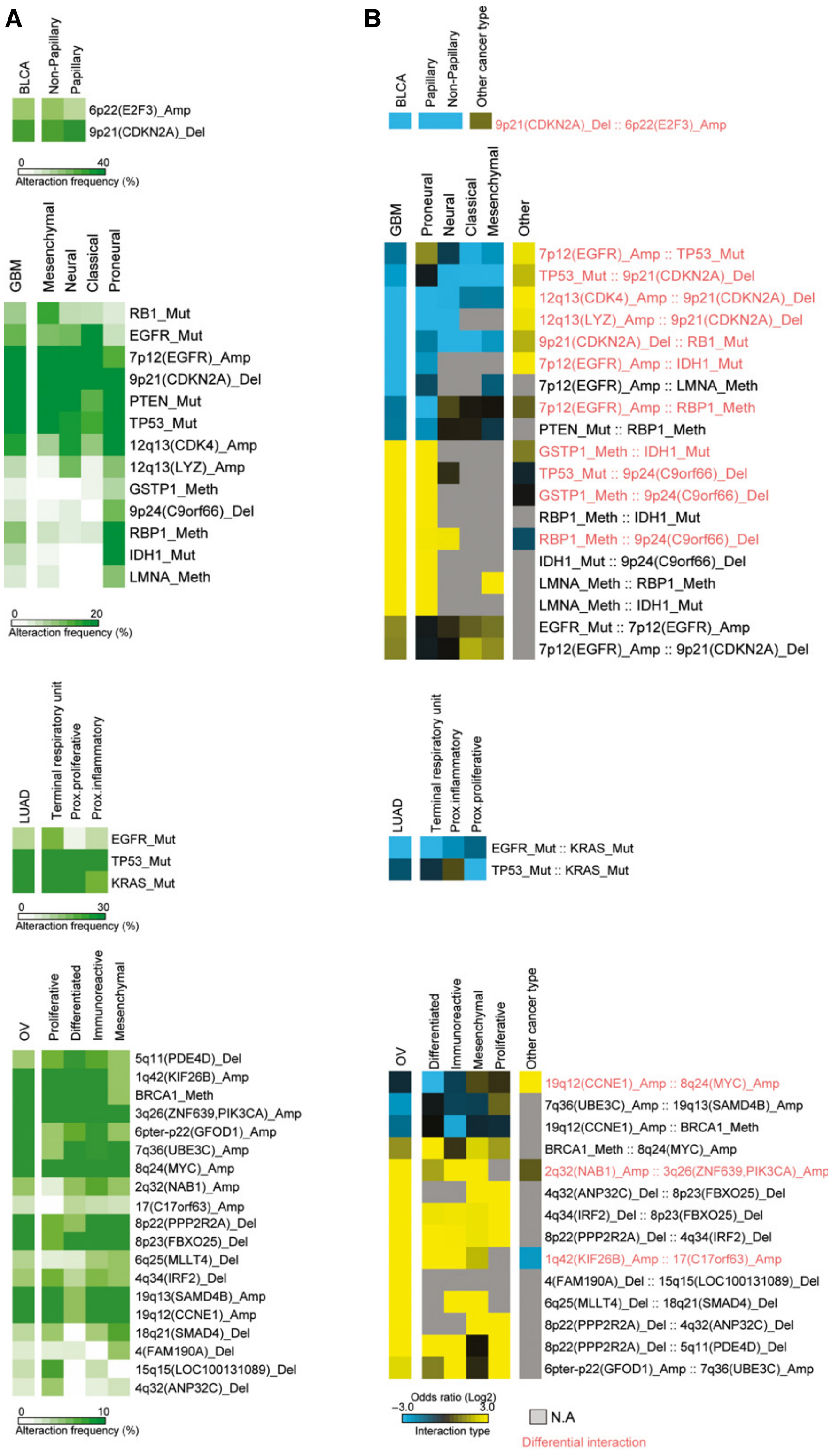

Figure EV5. Robustness of interactions across cancer types that were not considered in the main analysis.

A, B (A) Alteration frequencies of cancer genes across subtypes and (B) odds ratios of the detected interactions in cancer subtypes. Molecular subtypes are as previously defined (Cancer Genome Atlas Research Network, 2011, 2014a,b; Brennan *et al*, 2013).

© 2015 The Authors

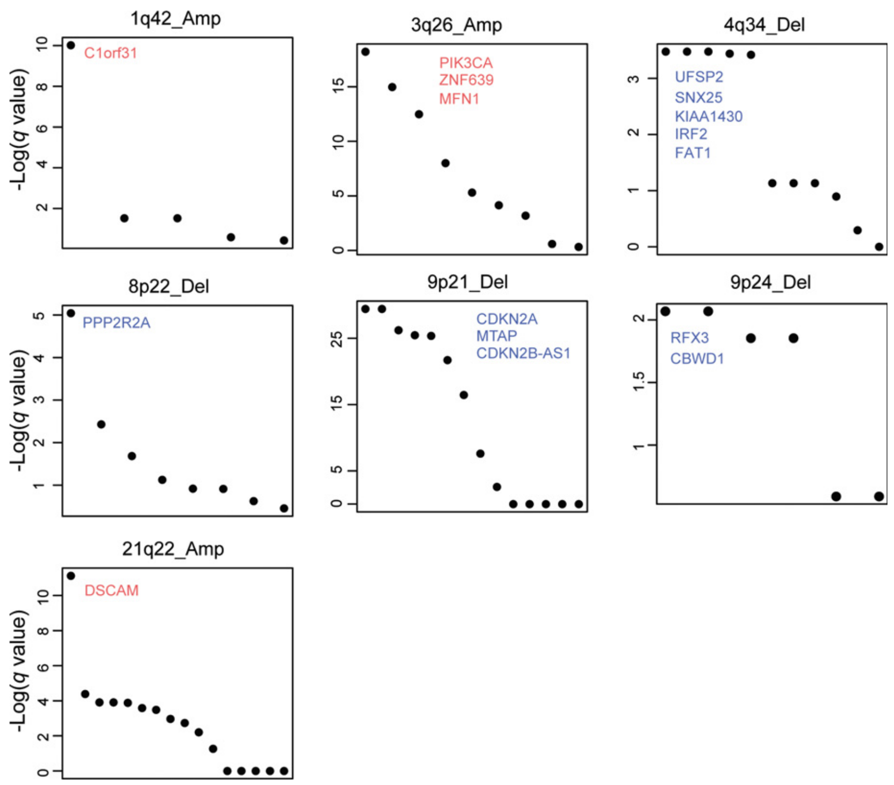

Figure EV6. Candidate driver genes within copy number alterations.

Seven recurrent gains and losses on chromosomes 1q, 3q, 4q, 8p, 9q, and 21q with differential interaction partners contain multiple genes. Genes are ranked within each copy number alteration using the FDR *q* values from a test for a difference in mRNA expression between altered and non-altered samples (Mann–Whitney test).

© 2015 The Authors
