# Supplementary material for: Cancer type-dependent genetic interactions between cancer driver alterations indicate plasticity of epistasis across cell types
Source: Mol Syst Biol. 2015 Jul 30;11(7):824. doi: 10.15252/msb.20156102 (PMC4547852; doi:10.15252/msb.20156102)
Supplement: Supplementary file 7 [file msb0011-0824-sd7.pdf]

Cancer-type dependent genetic interactions between cancer driver alterations indicate plasticity of epistasis across cell types

Solip Park and Ben Lehner

Corresponding author: Ben Lehner, Centre for Genomic Regulation, Barcelona

Review timeline:

Submission date:	15 February 2015
Editorial Decision:	21 April 2015
Revision received:	26 May 2015
Editorial Decision:	03 July 2015
Revision received:	07 July 2015
Accepted:	09 July 2015

Editor: Maria Polychronidou

Transaction Report:

(Note: With the exception of the correction of typographical or spelling errors that could be a source of ambiguity, letters and reports are not edited. The original formatting of letters and referee reports may not be reflected in this compilation.)

1st Editorial Decision 21 April 2015

Thank you again for submitting your work to Molecular Systems Biology. First of all I would like to apologize for the exceptional delay in getting back to you, which was due to the late arrival of one of the reports. We have now heard back from the three referees who agreed to evaluate your manuscript. As you will see from the reports below, the referees think that the presented findings seem interesting. However, they raise a series of concerns, which should be carefully addressed in a revision of the manuscript.

The reviewers' recommendations are clear and there is therefore no need to repeat the points listed below. In line with the comments of reviewer #2 we would ask you to include further discussion on the implications of the presented findings for improving cancer therapy.

Reviewer #1:

This paper considers the co-occurrence (positive epistatic interaction) or mutual exclusivity (negative epistatic interaction) of genetic events affecting gene function in human tumors. The authors define "driver alterations" as genes with mutation, copy number aberration, or hypermethylation in more than 2% of tumor samples, and identify pairs of positively and negatively interacting genes by comparing observed co-occurrence to a random model based on permutation.

The analytical approach represents an important contribution to the field of identifying genetic interaction/epistasis networks in cancer for functional genomics and drug targeting, and the conclusions of the report are particularly compelling. In particular, the statement:

© European Molecular Biology Organization

"...the plasticity of epistasis predicts that synthetic lethal strategies to kill cancer cells will often need to be specific for individual cancer types"

is a fundamentally important finding that, if true, will strongly impact the design, interpretation and generalizability of current and future genetic and chemogenetic screening projects in cell lines, xenografts and possibly tumor organoids.

The analysis of the identified interactions, however, is confusing, contradictory, and in some cases incorrect. A number of major points need to be addressed before this paper is suitable for publication:

1. P8 describes 55 interactions between 86 "driver alterations" when considering the entire tumor dataset, while P9 describes 60 interactions found when 22 tumor types are analyzed individually. What is the relationship between these two sets? Is the intersection enriched for positive or negative interactions? If there is enrichment, this plus the Venn diagram from Figure S1D might be better placed in Fig 1.

2. Figure 2A is confusing, in all aspects: the legend does not explain the data adequately and the text is also confusing. The first column sums to 62, which is in parens at the bottom, beside a cryptic "60." Does this imply that there are 60 unique interactions observed 62 times (i.e. Fig 1C)? This in turn implies that all but two of them are tissue specific interactions. If only 30/52 were confirmed in subsequent analyses (column 3), what is the explanation for the missed confirmations? Is there a relationship between confirmation by this method and detection in the pan-cancer analysis? Does the pan-cancer analysis actually only detect strong subtype-specific interactions?

3. The authors acknowledge that "unrecognized cancer subtypes" might cause false positive mutual exclusivity, but then argue that this is not a factor in their analysis because, among other reasons,

"...we detect a balanced number of co-occurrence and mutual exclusivity interactions, and both are observed to change across cancer types" (p14)

This is a logical flaw which is proven in the authors' data. Subtypes within one tumor type might be characterized by mutually exclusive mutations which show no such relationship in other types. To wit, Figure 3a shows the network of positive and negative interactions for GBM. If negative interactions define subtypes-the opposite of the authors' hypothesis-then the network decomposes into two primary clusters: IDH1-mutant and EGFR-amplification. These correspond perfectly to GBM subtypes discovered by genetic profiling (Verhaak et al., Cancer Cell 2010; see Figure 3 in particular).

4. Continuing with the data in Figure 3: this is the most data-rich figure in the paper, yet it merits only brief mention in the main text. A fuller description is warranted. Two points of clarification come to mind: first, what does "change in driver potency" mean? Does a big green dot, which corresponds to a "higher log odds ratio", mean the driver has higher potency in other types, or lower? Second, same issue with edges: does increased odds ratio difference (which is the opposite color-was that a good idea?) mean the interaction is more exclusive to the tumor type being studied, or more general across tumors? An explanation of an example tumor is warranted in the main text.

5. Figure 3, again: there is, by eye, a very strong correlation between red edges in panel A and yellow edges in panel C. Likewise, A/blue corresponds to C/green. Can you quantify this relationship? What is the biological basis of this? This reviewer strongly suspects it's driven by the subtypes.

Again, the data in Figure 3 is quite interesting, but it is a significant amount of work for the reader to figure out what the authors are really describing vis-‡-vis node size, edge width, line type, and color codes. A lot more effort needs to go into the text to justify the data and conclusions of Figure 3.

6. The concept of "driver potency" as defined by the authors conflates oncogenic potential with mutation frequency. The frequency of some (many?) driver mutations varies within the subtypes that the authors ignore; in fact subtypes are often defined based on these variations and covariations.

In addition, for genes with multiple interactions, the concept of potency is necessarily interaction-specific and does not count co-occurrence with other interaction partners.

Similarly, the described relationship between potency and interaction odds ratio is a tautology. Algebra will show that OR is a function of the reciprocal of the potency of the two genes; for genes with moderate (<<50%) mutation frequencies, an increase in the "potency" of either gene will result in a decrease in the observed odds ratio. The authors' use of the term "potency", despite the fact that they have defined this as the frequency of driver alterations in the methods, will be easily mis-interpreted and confuse many readers. I suggest simply using the term "frequency", as it properly identifies the quantity and has no functional connotation.

7. The idea of "proximal relationships in molecular interaction networks" (p. 11) needs additional granularity.

To summarize, the authors have used a rigorous method to identify pairs of genes that show co-occurring or mutually exclusive modifications in cancer genomes. The interpretation of the resulting network should be refined. The primary weakness seems to be the acceptance of subtypes within the tumor types. It is no bad thing that subtypes might drive these positive and negative epistatic interactions; in fact co-occurring, subtype-specific variations may be more clinically relevant than those same interactions without subtype information.

Minor points:

1. Heading on p10 makes no sense. Typo?

2. A -log10 scale on the Y-axis would be more intuitive.

3. Figure 3 is ref'd in the main text before Figure 2 (p10)

4. Fig 2d: "integrated" PPIs

5. p 17 2nd paragraph. Clarify the first sentence.

Reviewer #2:

Summary.
In this manuscript the authors interrogate co-occurrence and mutual exclusivity of cancer driver mutations across various tumor types using publicly available data derived from some 3000 tumor samples. The main motivation of this is to investigate if and how these genetic interactions change in different epigenetic contexts. They show that epistatic interactions are highly dependent on the tissue origin and that most interactions are in fact private to a single tumor type. Furthermore, they find an inverse correlation between driver potency (defined as specific mutational frequency) and the number of detected co-operating mutations.

General remarks
This is a well-written and executed manuscript on an important topic i.e., gene-gene interactions and their genetic and epigenetic context dependency. The strength of the paper is that it is a comprehensive analysis of co-occurrence and mutual exclusivity in cancer. The analysis is thorough and the display of the interaction networks and changes when compared to other tissues (Figure 3) is convincing and provides a nice graphical illustration of the extensive rewiring.

Improved understanding of epistasis in cancer may improve the application of existing drugs and allow the development of novel agents, particularly those based on synthetic lethality principles. This is also stated in the abstract as one of the major areas where the findings of the paper would have an impact. One conceptual difficulty with this extrapolation is that the interactions that are investigated here actually do not directly relate to synthetic lethality. Driver mutation co-occurrence indicates cooperativity, whereas mutual exclusivity typically reflects redundancy, only in rare cases will it reflect synthetic lethality. This does not mean that the architectural principles of cancer driver mutation networks are fundamentally different. But one should be cautious or at least explicit about

© European Molecular Biology Organization

this limitation of the approach taken here and its implications of SL therapy.

A second aspect that limits my enthusiasm for this paper is that the notion that epistatic interactions are highly tissue specific is not surprising. It is very well known that driver mutation frequencies differ dramatically between tumor types and from model organisms it is well known that genetic interaction networks show extensive rewiring depending on context or species. Hence, it would have been very surprising indeed if genetic interactions in cancer would display little context dependency. Furthermore, it is not clear to me how one would employ the methodology or uncovered interactions for the development of novel biomarkers or therapeutics. Just knowing that interactions are context dependent does not help in making better therapeutics, it only highlights the problem. The notion that strong drivers have fewer co-occurring interactions is interesting but also expected.

The method for detection co-occurance/exclusivity is well executed but not new (e.g. Ciriello et al 2012). The conceptual advance is limited. The new interactions are not further investigated experimentally nor do they provide immediate direction for improving cancer therapy. Therefore, and despite the nice job on making the interaction maps, I struggle to identify the "discovery" in this paper.

Minor comments and suggestions
Given the importance of the gene interactions in cancer, it would be good to extend the presented interaction map to provide practical guidance for future computational or experimental work. (Specific questions / suggestions below).

Figure 1

Panel B only shows 14 cancer types, not 22. It is unclear whether the remaining cancer types are omitted because they do not have any interactions or because the analysis is underpowered. (Why is COADREAD_MSS omitted?)

Why are there so many interaction in GBM? Is it because GBM is more heterogeneous or because there are more samples in that cohort? Is there a trend between heterogeneity and number of interactions?

Given that the authors suggest that many more interactions will be discovered as more tumors are sequenced, how would panel B look with e.g. twice as much data as currently? An answer to this question may offer a very concrete target or a warning to further cohort studies of mutations in cancer.

Given the saturation analysis, how would the cancer types compare if the cohorts were of equal size?
[For sure there would still be differences between the cancer types - KIRC has a huge cohort and few interactions. But other combinations are not clear, e.g. LUSC vs OV]

Figure 4

Panel A is rather confusing. The text refers to a "change in interaction" axis, but the plot has potencies on both axis. Given that the panel shows pairwise interactions, why is the scatter not symmetrical?

In panel B, one gene pair contributes multiple points to the diagram (each interaction is compared to multiple tissues). Furthermore, a frequent driver gene may contributes to multiple gene pairs. Each dot is therefore not entirely independent. How is this controlled for? In other words, it is hard to judge whether the trend is a general property of cancer genes or whether it is driven by one or two genes, for example with tissue-specific expression.

Figure 5

The text in section "Interpreting the coupling..." is clear enough, but its link to Figure 5 is cryptic/unlabeled. Does the text refer to cross-talk in panels B and C?

Materials and Methods

The definition of driver potency is troubling. The name "potency" (and the manuscript text) suggests that it is a fixed property for a gene A (at least within one cancer/tissue type). However, equation on page 26 suggests that it is conditional on a second alteration of a gene B. This can lead to confusion. Consider for example three possible mutated genes - X, Y, and Z - with the following frequencies:

X only - 20
Y only - 0
Z only - 60
XY not Z - 20
other combinations - 0
none - 0

Using Y as the second alteration, the potency of gene X would be 20 / (20 + 60) = 0.25
Using Z as the second alteration, the potency of gene X would be 20 / (20 + 0) = 1
(Alternatively, one could interpret the definition of "Aonly" in the equation as "containing A but not the second alteration", in which case the potency of X would be (20 + 20) / (20+ 20 + 0) = 1)

Reviewer #3:

It is well established that the genetic alterations that drives cancer often interact epistatically, and in some cases it has been shown that epistatic interactions are confined to a certain tumor type. Park et al. performed a comprehensive analysis of epistatic relationships in >3,000 cancer samples from the TCGA project, analyzing co-occurrence and mutual exclusivity relationships between driver alterations in more than three thousand human tumors.

Using a carefully controlled statistical analysis the authors showed that 57% of the interactions were specific to particular types of cancer. They exclude intra-tumor heterogeneity and unrecognized cancer subtypes as confounders driving a significant portion of the signal. As genomic alterations make more significant mutual exclusivity interactions and fewer co-occurrence interactions they become more potent drivers of cancer. Hence, the authors provide convincing evidence that more than half of the interactions between cancer drivers are specific to particular types of cancer, a finding with important implications for understanding tumor biology and also for exploitation of synthetic lethality in cancer therapy.

A number of elegant analyses have been presented clearly in this manuscript. I have several suggestions that may clarify specific aspects of the paper but I don't believe that any additional experiments or difficult analyses are needed.

- Interestingly, the interactions showing cancer type-specificity are more enriched between genes encoding physically or functionally interacting proteins than interactions detected when considering all cancer types together (pan-cancer analysis). Does this imply that the pan-cancer analysis is 'noisier' than the tumor-type-specific analysis in this regard, i.e. is this analysis more prone to identify 'spurious epistatis events' stemming from the fact that one cancer type shows mostly mutation 'X 'and the other tumor type mostly mutation 'Y'.

- The authors findings suggest that at least half of the interactions between cancer drivers are specific to particular types of cancer. To what extent is this difference/variation observable in different cancer subtypes (arising in the same tissue) vs. cancers that arise in different organs? Are there differences in the relative level at which the interactions between cancer drivers are specific or preserved across cancer types vs. across cancer subtypes?

- The authors state that their analyses have an important take-home message for the exploitation of synthetic lethality in cancer therapy, predicting that particular synthetic lethal strategies will often only prove effective in a limited subset of cancers carrying a targeted vulnerability. I agree that the works by Ashworth are relevant to this, but suggest works by Bernards (e.g. on strategies for treating colon cancer through jointly targeting BRAF and EGFR; PMID:22281684) could also be cited in this context.

- Lastly, while overall nicely written I feel that at parts there is some redundancies in the text that could be shortened to make the text more concise, e.g. text relating to the interpretation of the coupling between changes in interaction and changes in driver potency.

1st Revision - authors' response 26 May 2015

Thank you for the helpful suggestions. Please see below for the modifications we have made in response to each of the referees' comments. As requested by the editor, we have also shortened the abstract. Additions to the text are marked in green.

Reviewer #1:

This paper considers the co-occurrence (positive epistatic interaction) or mutual exclusivity (negative epistatic interaction) of genetic events affecting gene function in human tumors. The authors define "driver alterations" as genes with mutation, copy number aberration, or hypermethylation in more than 2% of tumor samples, and identify pairs of positively and negatively interacting genes by comparing observed co-occurrence to a random model based on permutation.

The analytical approach represents an important contribution to the field of identifying genetic interaction/epistasis networks in cancer for functional genomics and drug targeting, and the conclusions of the report are particularly compelling. In particular, the statement:

"...the plasticity of epistasis predicts that synthetic lethal strategies to kill cancer cells will often need to be specific for individual cancer types"

is a fundamentally important finding that, if true, will strongly impact the design, interpretation and generalizability of current and future genetic and chemogenetic screening projects in cell lines, xenografts and possibly tumor organoids.

We thank the referee for his/her enthusiastic and positive evaluation of the manuscript.

The analysis of the identified interactions, however, is confusing, contradictory, and in some cases incorrect. A number of major points need to be addressed before this paper is suitable for publication:

1. P8 describes 55 interactions between 86 "driver alterations" when considering the entire tumor dataset, while P9 describes 60 interactions found when 22 tumor types are analyzed individually. What is the relationship between these two sets? Is the intersection enriched for positive or negative interactions? If there is enrichment, this plus the Venn diagram from Figure S1D might be better placed in Fig 1.

22 interactions were detected in both the pan-cancer and single cancer analyses (Revised Fig 1C). These interactions are evenly balanced between positive (N = 10) and negative (N = 11) interactions (Revised Fig 1C).

2. Figure 2A is confusing, in all aspects: the legend does not explain the data adequately and the text is also confusing.

We apologize for the confusion – we have expanded the legend to clarify what the numbers mean.

The first column sums to 62, which is in parens at the bottom, beside a cryptic "60." Does this imply that there are 60 unique interactions observed 62 times (i.e. Fig 1C)? This in turn implies that all but two of them are tissue specific interactions.

Yes. We detected 60 unique interactions across cancer types and two of them were detected in more than one cancer type as shown in Revised Fig 1D.

If only 30/52 were confirmed in subsequent analyses (column 3), what is the explanation for the missed confirmations? Is there a relationship between confirmation by this method and detection in the pan-cancer analysis? Does the pan-cancer analysis actually only detect strong subtype-specific interactions?

21 of 52 interactions (40%) were also detected in the pan-cancer analysis and 9 of them were score as differential interactions comparing between cancer types (30%). We observed that the overlapping number of interactions between pan-cancer analysis and differential interactions is non-significant ($P = 0.093$, Fisher's exact test).

3. The authors acknowledge that "unrecognized cancer subtypes" might cause false positive mutual exclusivity, but then argue that this is not a factor in their analysis because, among other reasons,

"...we detect a balanced number of co-occurrence and mutual exclusivity interactions, and both are observed to change across cancer types" (p14)

This is a logical flaw which is proven in the authors' data. Subtypes within one tumor type might be characterized by mutually exclusive mutations which show no such relationship in other types. To wit, Figure 3a shows the network of positive and negative interactions for GBM. If negative interactions define subtypes-the opposite of the authors' hypothesis-then the network decomposes into two primary clusters: IDH1-mutant and EGFR-amplification. These correspond perfectly to GBM subtypes discovered by genetic profiling (Verhaak et al., Cancer Cell 2010; see Figure 3 in particular).

We checked for the effects of cancer subtypes in two ways. First, in the main analysis all of the randomisations were performed within each subtype defined by TCGA (Ciriello et al, Nature Genetics, 2013). The enrichment or depletion of driver alterations in a TCGA subtype therefore cannot account for any interactions. Second, we analysed the detected interactions across subtypes defined in additional papers but not explicitly controlled for in our randomisation procedure (Supplementary Figure 6). For example, for GBM (panel copied below) we used the subtypes defined in the updated analysis of Brennan CW et al., Cell 2013. The interactions detected in the complete GBM dataset can still be observed in one or more of the subtypes. The interactions are not an artefact of the enrichment of particular drivers in particular subtypes. However, some interactions may indeed not be detected in all subtypes of a cancer.

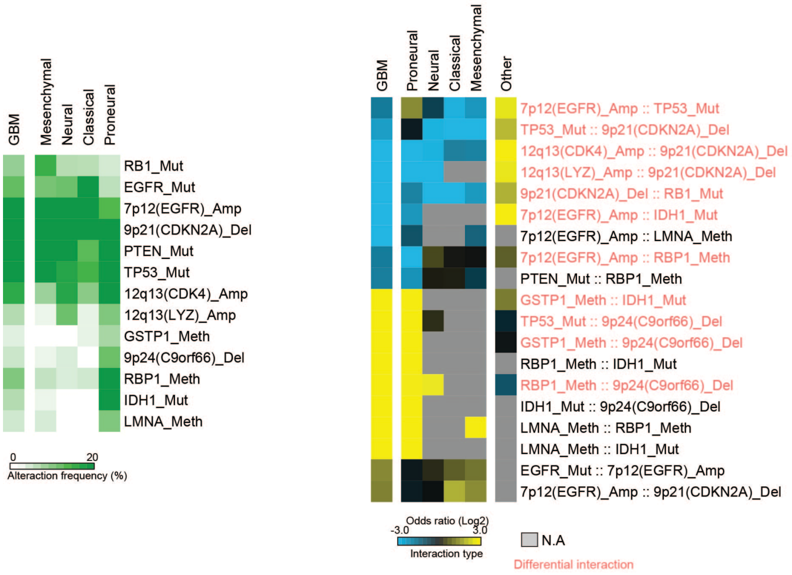

4. Continuing with the data in Figure 3: this is the most data-rich figure in the paper, yet it merits only brief mention in the main text. A fuller description is warranted. Two points of clarification come to mind: first, what does "change in driver potency" mean? Does a big green dot, which

corresponds to a "higher log odds ratio", mean the driver has higher potency in other types, or lower? Second, same issue with edges: does increased odds ratio difference (which is the opposite color-was that a good idea?) mean the interaction is more exclusive to the tumor type being studied, or more general across tumors? An explanation of an example tumor is warranted in the main text.

We have simplified figure 3 in the revised manuscript and expanded the legend.

5. Figure 3, again: there is, by eye, a very strong correlation between red edges in panel A and yellow edges in panel C. Likewise, A/blue corresponds to C/green. Can you quantify this relationship? What is the biological basis of this? This reviewer strongly suspects it's driven by the subtypes.

This is correct (see figure below, r = 0.89), but it is a trivial result of the analysis – if a strong positive interaction between two drivers is detected in one cancer type and the interaction does not occur in another tissue, then the odds ratio difference will be positive and strong. The converse is also true: if a negative interaction is detected between two drivers in one cancer type but not in another – the odds ratio difference will be negative and strong.

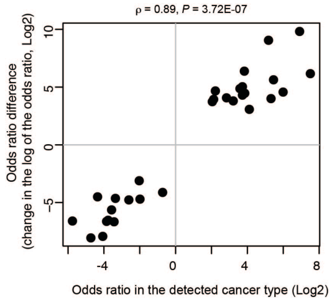

Again, the data in Figure 3 is quite interesting, but it is a significant amount of work for the reader to figure out what the authors are really describing vis-à-vis node size, edge width, line type, and color codes. A lot more effort needs to go into the text to justify the data and conclusions of Figure 3.

We have simplified Figure 3 and expanded the figure legend to better explain the presented data.

6. The concept of "driver potency" as defined by the authors conflates oncogenic potential with mutation frequency. The frequency of some (many?) driver mutations varies within the subtypes that the authors ignore; in fact subtypes are often defined based on these variations and covariations. In addition, for genes with multiple interactions, the concept of potency is necessarily interaction-specific and does not count co-occurrence with other interaction partners.

Similarly, the described relationship between potency and interaction odds ratio is a tautology. Algebra will show that OR is a function of the reciprocal of the potency of the two genes; for genes with moderate (<<50%) mutation frequencies, an increase in the "potency" of either gene will result in a decrease in the observed odds ratio. The authors' use of the term "potency", despite the fact that they have defined this as the frequency of driver alterations in the methods, will be easily mis-interpreted and confuse many readers. I suggest simply using the term "frequency", as it properly identifies the quantity and has no functional connotation.

In the revised manuscript we have removed the analysis of frequency/potency.

7. The idea of "proximal relationships in molecular interaction networks" (p. 11) needs additional granularity.

Sentence deleted (previous sentences are more precise).

To summarize, the authors have used a rigorous method to identify pairs of genes that show co-

© European Molecular Biology Organization

occurring or mutually exclusive modifications in cancer genomes. The interpretation of the resulting network should be refined. The primary weakness seems to be the acceptance of subtypes within the tumor types. It is no bad thing that subtypes might drive these positive and negative epistatic interactions; in fact co-occurring, subtype-specific variations may be more clinically relevant than those same interactions without subtype information.

Minor points:

1. Heading on p10 makes no sense. Typo?

Changed to: "Interactions between cancer drivers are frequently cancer type-specific"

2. A -log10 scale on the Y-axis would be more intuitive.

Changed (revised Fig2B).

3. Figure 3 is ref'd in the main text before Figure 2 (p10)

Reference removed – Supplementary Table 4 is the correct reference.

4. Fig 2d: "integrated" PPIs

Thanks for the correction – we have changed this.

5. p 17 2nd paragraph. Clarify the first sentence.

Re-phrased as: "We found that at least half of the interactions between cancer drivers differ in the strength of interaction in different cancer types. This suggests that how genomic alterations interact cooperatively or partially redundantly to driver cancer varies substantially in different cancers."

Reviewer #2:

Summary.
In this manuscript the authors interrogate co-occurrence and mutual exclusivity of cancer driver mutations across various tumor types using publicly available data derived from some 3000 tumor samples. The main motivation of this is to investigate if and how these genetic interactions change in different epigenetic contexts. They show that epistatic interactions are highly dependent on the tissue origin and that most interactions are in fact private to a single tumor type. Furthermore, they find an inverse correlation between driver potency (defined as specific mutational frequency) and the number of detected co-operating mutations.

General remarks
This is a well-written and executed manuscript on an important topic i.e., gene-gene interactions and their genetic and epigenetic context dependency. The strength of the paper is that it is a comprehensive analysis of co-occurrence and mutual exclusivity in cancer. The analysis is thorough and the display of the interaction networks and changes when compared to other tissues (Figure 3) is convincing and provides a nice graphical illustration of the extensive rewiring.

We thank the referee for his/her enthusiastic and positive evaluation of the manuscript.

Improved understanding of epistasis in cancer may improve the application of existing drugs and allow the development of novel agents, particularly those based on synthetic lethality principles. This is also stated in the abstract as one of the major areas where the findings of the paper would have an impact. One conceptual difficulty with this extrapolation is that the interactions that are investigated here actually do not directly relate to synthetic lethality. Driver mutation co-occurrence indicates cooperativity, whereas mutual exclusivity typically reflects redundancy, only in rare cases will it reflect synthetic lethality. This does not mean that the architectural principles of cancer driver

© European Molecular Biology Organization

mutation networks are fundamentally different. But one should be cautious or at least explicit about this limitation of the approach taken here and its implications of SL therapy.

We agree and have edited this sentence in the abstract to read: “In addition, if this plasticity of epistasis across cell types is also true for synthetic lethal interactions, a synthetic lethal strategy to kill cancer cells may frequently work in one cancer type but prove ineffective in a second.” Similarly in the discussion: “If the plasticity of epistasis that we detected here is also true for synthetic lethal interactions, then our results...”

A second aspect that limits my enthusiasm for this paper is that the notion that epistatic interactions are highly tissue specific is not surprising. It is very well known that driver mutation frequencies differ dramatically between tumor types and from model organisms it is well known that genetic interaction networks show extensive rewiring depending on context or species. Hence, it would have been very surprising indeed if genetic interactions in cancer would display little context dependency. Furthermore, it is not clear to me how one would employ the methodology or uncovered interactions for the development of novel biomarkers or therapeutics. Just knowing that interactions are context dependent does not help in making better therapeutics, it only highlights the problem. The notion that strong drivers have fewer co-occurring interactions is interesting but also expected.

The method for detection co-occurance/exclusivity is well executed but not new (e.g. Ciriello et al 2012). The conceptual advance is limited. The new interactions are not further investigated experimentally nor do they provide immediate direction for improving cancer therapy. Therefore, and despite the nice job on making the interaction maps, I struggle to identify the "discovery" in this paper.

We agree that the results are consistent with the environmental context-dependence of epistasis in unicellular organisms. However we do think that if you told most cancer biologists that two driver alterations co-operate in one cancer type they would also expect them to co-operate in another cancer type i.e. in our experience researchers tend to think by default that cancer genes co-operate or act redundantly in the same way in different cell types. This is a reasonable first assumption, but the data indicate that it is frequently wrong. We would not claim that our study has any immediate therapeutic implications (although possibly it should influence how e.g. multi-cancer drug trials are designed or analysed – the cell type-specificity of how drivers work will likely mean drugs effective in one type of cancer carrying a defined mutation will not be effective in a second type of cancer). More importantly, we think that the study demonstrates a basic feature of genetic architecture in multicellular organisms and one that has important implications for understanding evolution and disease genetics in humans.

Minor comments and suggestions
Given the importance of the gene interactions in cancer, it would be good to extend the presented interaction map to provide practical guidance for future computational or experimental work. (Specific questions / suggestions below).

Figure 1

Panel B only shows 14 cancer types, not 22. It is unclear whether the remaining cancer types are omitted because they do not have any interactions or because the analysis is underpowered. (Why is COADREAD_MSS omitted?)

The remaining 8 cancer types are omitted because we didn’t detect any interactions. This is now also stated in the figure legend.

Why are there so many interactions in GBM? Is it because GBM is more heterogeneous or because there are more samples in that cohort? Is there a trend between heterogeneity and number of interactions?

Across the cancer types there is only a marginally significant correlation between the number of analysed samples and the number of detected interactions (Spearman correlation = 0.52, *P*-value = 0.0549). There isn’t a significant correlation between the number of driver alterations tested in a

cancer type and the number of detected interactions (Spearman correlation = 0.09, *P*-value = 0.762). However, there is a significant correlation between the median number of samples in which the tested driver alterations occur in a cancer type and the number of detected interactions (Spearman correlation = 0.64, *P*-value = 0.0013). These analyses are presented in Revised Supplementary Figure 2 and referred to in the results section of the text.

Given that the authors suggest that many more interactions will be discovered as more tumors are sequenced, how would panel B look with e.g. twice as much data as currently? An answer to this question may offer a very concrete target or a warning to further cohort studies of mutations in cancer.

Although it is dangerous to extrapolate trends, we present in Fig. S1 E-G sub-sampling analyses that suggest the number of detected interactions is ~linearly increasing as the number of samples is increased in both individual cancer types and in the pan-cancer analysis.

Given the saturation analysis, how would the cancer types compare if the cohorts were of equal size?
[For sure there would still be differences between the cancer types - KIRC has a huge cohort and few interactions. But other combinations are not clear, e.g. LUSC vs OV]

Please see the response above – as shown in Supplementary Figure 2, there is a reasonable correlation between the median number of samples in which the tested driver alterations occur and the number of detected interactions.

Figure 4

Panel A is rather confusing. The text refers to a "change in interaction" axis, but the plot has potencies on both axis. Given that the panel shows pairwise interactions, why is the scatter not symmetrical?

In panel B, one gene pair contributes multiple points to the diagram (each interaction is compared to multiple tissues). Furthermore, a frequent driver gene may contributes to multiple gene pairs. Each dot is therefore not entirely independent. How is this controlled for? In other words, it is hard to judge whether the trend is a general property of cancer genes or whether it is driven by one or two genes, for example with tissue-specific expression.

We apologize for the confusion. Please refer to the reply to comment #6 of reviewer 1. Now we have removed the analyses of potency from the revised manuscript (related with Figure 4 and 5).

Figure 5
The text in section "Interpreting the coupling..." is clear enough, but its link to Figure 5 is cryptic/unlabeled. Does the text refer to cross-talk in panels B and C?
Materials and Methods
The definition of driver potency is troubling. The name "potency" (and the manuscript text) suggests that it is a fixed property for a gene A (at least within one cancer/tissue type). However, equation on page 26 suggests that it is conditional on a second alteration of a gene B. This can lead to confusion. Consider for example three possible mutated genes - X, Y, and Z - with the following frequencies:
X only - 20
Y only - 0
Z only - 60
XY not Z - 20
other combinations - 0
none - 0
Using Y as the second alteration, the potency of gene X would be 20 / (20 + 60) = 0.25
Using Z as the second alteration, the potency of gene X would be 20 / (20 + 0) = 1
(Alternatively, one could interpret the definition of "Aonly" in the equation as "containing A but not the second alteration", in which case the potency of X would be (20 + 20) / (20+ 20 + 0) = 1)

We have removed the analyses of potency from the revised manuscript.

Reviewer #3:

It is well established that the genetic alterations that drives cancer often interact epistatically, and in some cases it has been shown that epistatic interactions are confined to a certain tumor type. Park et al. performed a comprehensive analysis of epistatic relationships in >3,000 cancer samples from the TCGA project, analyzing co-occurrence and mutual exclusivity relationships between driver alterations in more than three thousand human tumors.

Using a carefully controlled statistical analysis the authors showed that 57% of the interactions were specific to particular types of cancer. They exclude intra-tumor heterogeneity and unrecognized cancer subtypes as confounders driving a significant portion of the signal. As genomic alterations make more significant mutual exclusivity interactions and fewer co-occurrence interactions they become more potent drivers of cancer. Hence, the authors provide convincing evidence that more than half of the interactions between cancer drivers are specific to particular types of cancer, a finding with important implications for understanding tumor biology and also for exploitation of synthetic lethality in cancer therapy.

A number of elegant analyses have been presented clearly in this manuscript. I have several suggestions that may clarify specific aspects of the paper but I don't believe that any additional experiments or difficult analyses are needed.

We thank the referee for his/her enthusiastic and positive evaluation of the manuscript.

- Interestingly, the interactions showing cancer type-specificity are more enriched between genes encoding physically or functionally interacting proteins than interactions detected when considering all cancer types together (pan-cancer analysis). Does this imply that the pan-cancer analysis is 'noisier' than the tumor-type-specific analysis in this regard, i.e. is this analysis more prone to identify 'spurious epistatis events' stemming from the fact that one cancer type shows mostly mutation 'X 'and the other tumor type mostly mutation 'Y'.

We don't have a good suggestion for this, however in the pan-cancer analysis we also randomised the data within cancer (sub)types to control for the heterogeneous distribution of alterations across cancer (sub)types, so this is not the explanation. The important result is that the individual cancer interactions are not less biologically meaningful than the pan-cancer interactions.

- The authors findings suggest that at least half of the interactions between cancer drivers are specific to particular types of cancer. To what extent is this difference/variation observable in different cancer subtypes (arising in the same tissue) vs. cancers that arise in different organs? Are there differences in the relative level at which the interactions between cancer drivers are specific or preserved across cancer types vs. across cancer subtypes?

For the interactions detected in the 3 tissues where we considered subtypes (i.e. BRCA, COADREAD, UCEC), we tested 9 interactions a total of 37 times in different cancers (or in subtypes of different cancers) and detected 7/9 interactions as differential in at least one comparison (13/37 tests, 35%). We tested 11 interactions 27 times in different subtypes of cancer from the same tissue and detected 4/11 as differential (5/27 tests, 19%). There is no significant difference in the number of interactions detected in the within tissue vs. between tissue comparisons ($P = 0.17$, Fisher's exact test). This data is presented in Supplementary Fig.4.

- The authors state that their analyses have an important take-home message for the exploitation of synthetic lethality in cancer therapy, predicting that particular synthetic lethal strategies will often only prove effective in a limited subset of cancers carrying a targeted vulnerability. I agree that the works by Ashworth are relevant to this, but suggest works by Bernards (e.g. on strategies for treating colon cancer through jointly targeting BRAF and EGFR; PMID:22281684) could also be cited in this context.

We agree and have added the reference.

- Lastly, while overall nicely written I feel that at parts there is some redundancies in the text that could be shortened to make the text more concise, e.g. text relating to the interpretation of the coupling between changes in interaction and changes in driver potency.

We have removed this text from the revised manuscript.

2nd Editorial Decision 03 July 2015

Thank you again for submitting your work to Molecular Systems Biology. We have now heard back from the referee who agreed to evaluate your manuscript. As you will see below, the referee is satisfied with the modifications made and thinks that the study is now suitable for publication.

Before formally accepting the manuscript, we would ask you to address some minor editorial issues listed below.

Reviewer #2:

My main concern with the paper was the practical usability of the interaction network for cancer therapies. By toning down the speculations and conclusions in the text this has been sufficiently addressed. This remains an interesting study.

I still don't really think that most biologists would assume that genetic interactions are strongly conserved between cell types but this is only a minor disagreement and it certainly does not harm to point this out once more.

© European Molecular Biology Organization
